# Immobilization of carboxypeptidase from *Sulfolobus solfataricus* on magnetic nanoparticles improves enzyme stability and functionality in organic media

**DOI:** 10.1186/1472-6750-14-82

**Published:** 2014-09-05

**Authors:** Silvia Sommaruga, Elisabetta Galbiati, Jesus Peñaranda-Avila, Chiara Brambilla, Paolo Tortora, Miriam Colombo, Davide Prosperi

**Affiliations:** 1 Dipartimento di Biotecnologie e Bioscienze, Università di Milano-Bicocca, Piazza della Scienza 2, Milano 20126, Italy; 2 Istituto di Scienze e Tecnologie Molecolari, CNR, via Fantoli 16/15, Milano 20138, Italy

**Keywords:** Carboxypeptidase, Magnetic nanoparticles, His-tag immobilization, Enzyme stability, Hyperthermophilic microorganisms

## Abstract

**Background:**

Superparamagnetic iron oxide nanoparticles (MNP) offer several advantages for applications in biomedical and biotechnological research. In particular, MNP-based immobilization of enzymes allows high surface-to-volume ratio, good dispersibility, easy separation of enzymes from the reaction mixture, and reuse by applying an external magnetic field. In a biotechnological perspective, extremophilic enzymes hold great promise as they often can be used under non-conventional harsh conditions, which may result in substrate transformations that are not achievable with normal enzymes. This prompted us to investigate the effect of MNP bioconjugation on the catalytic properties of a thermostable carboxypeptidase from the hyperthermophilic archaeon *Sulfolobus solfataricus* (CP*Sso*), which exhibits catalytic properties that are useful in synthetic processes.

**Results:**

CPSso was immobilized onto silica-coated iron oxide nanoparticles via NiNTA-His tag site-directed conjugation. Following the immobilization, CP*Sso* acquired distinctly higher long-term stability at room temperature compared to the free native enzyme, which, in contrast, underwent extensive inactivation after 72 h incubation, thus suggesting a potential utilization of this enzyme under low energy consumption. Moreover, CP*Sso* conjugation also resulted in a significantly higher stability in organic solvents at 40°C, which made it possible to synthesize N-blocked amino acids in remarkably higher yields compared to those of free enzyme.

**Conclusions:**

The nanobioconjugate of CPSso immobilized on silica-coated magnetic nanoparticles exhibited enhanced stability in aqueous media at room temperature as well as in different organic solvents. The improved stability in ethanol paves the way to possible applications of immobilized CP*Sso*, in particular as a biocatalyst for the synthesis of N-blocked amino acids. Another potential application might be amino acid racemate resolution, a critical and expensive step in chemical synthesis.

## Background

Due to their unique magnetic properties, superparamagnetic iron oxide nanoparticles (MNP) represent a well-known and effective nanosystem for applications in biomedical and biotechnological research. In fact, MNP are being developed as drug carriers [1,2], imaging agents [1,3], analytical probes [4,5] and recyclable support for enzyme immobilization [6,7].

Compared with chemical catalysis, enzyme-mediated reactions allow for more specific chemo-, regio-, and stereo-selectivity in organic synthesis [8]. However, long-term stability and recyclability of enzymes have been considered the main limitations to their extensive utilization [9].

Recently, several nanoparticles have been employed to improve traditional enzyme immobilization methods in order to enhance loading, activity and stability of enzymes and to reduce the biocatalyst costs in industrial biotechnology [10,11]. In particular, MNP-based immobilization of enzymes presents several advantages, including (i) high surface-to-volume ratio offered by nanosize magnetic beads, (ii) good dispersibility, (iii) easy separation of enzymes from the reaction mixture, and (iv) reuse by applying an external magnetic field [12]. One of the crucial points in protein immobilization on nanoscale solid surfaces is that structural modifications may occur, which could affect protein activity and stability to different extents depending on the protein and the conjugation strategy [13]. For this reason, there has been an increasing interest in developing new reliable approaches for the immobilization of enzymes on magnetic nanoparticles [14-16]. However, although great efforts have been made for this purpose, the actual effect of immobilization on enzyme functionality is still poorly understood. Furthermore, no such studies involving enzymes from extremophile microorganisms have been carried out so far.

Extremophilic enzymes hold great promise in industrial biotechnology, as they can often be used under non-conventional harsh conditions, which may result in substrate transformations that are not achievable with normal enzymes [17].

In this work, we investigated the effect of MNP bioconjugation on the catalytic properties of a thermostable carboxypeptidase from *Sulfolobus solfataricus* (CP*Sso*). CP*Sso* is a heat- and pressure-resistant zinc-metalloprotease consisting of four identical 43 kDa subunits [17-20]. The catalytic and kinetic mechanisms of CP*Sso* have been well established and were confirmed by a 3D model that was developed and validated in the past years [21,22]. CP*Sso* exhibits nonconventional catalytic properties that are useful in several synthetic processes. First, it removes any amino acid from the C-terminus of short peptides, with the sole exception of proline, and also hydrolyzes N-blocked amino acids, thus acting as an aminoacylase [22]. Second, despite its remarkable thermophilicity, it maintains a significant fraction of its maximal activity even at room temperature. Finally, CP*Sso* maintains a significant activity in solvent mixtures even at high content of organic fraction [23]. These peculiar properties highlight the biotechnological potential of this enzyme, in particular to achieve the synthesis of N-blocked amino acid in organic media.

## Results

### Synthesis of Ni^2+^-functionalized silica-coated magnetic nanoparticles (NiNTASiMNP)

**Figure 1:**
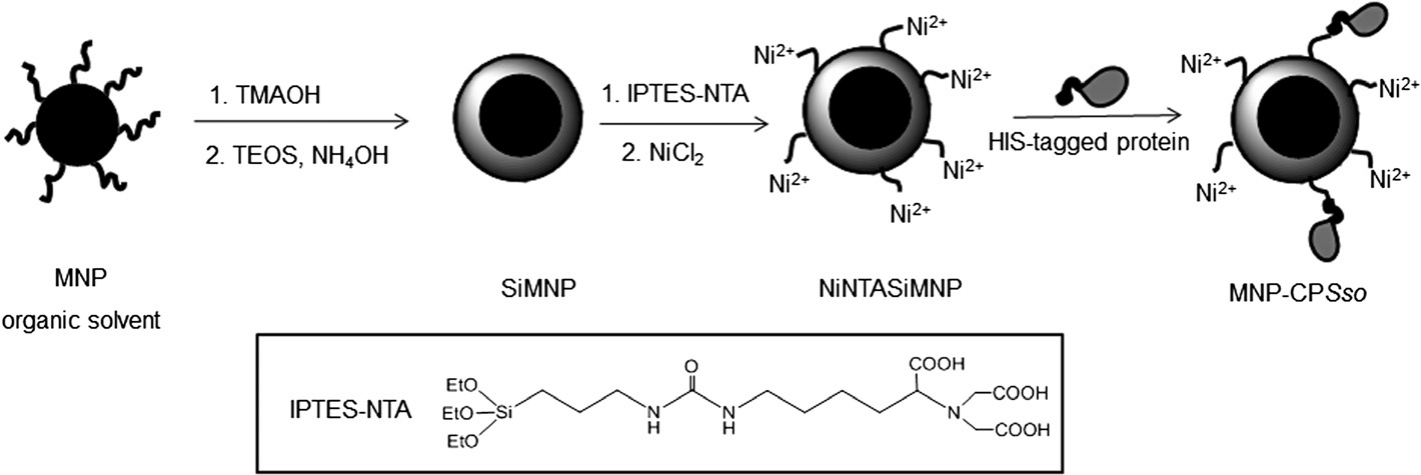
**Procedure of CP** ***Sso *** **immobilization on NiNTASiMNP.**

**Figure 2:**
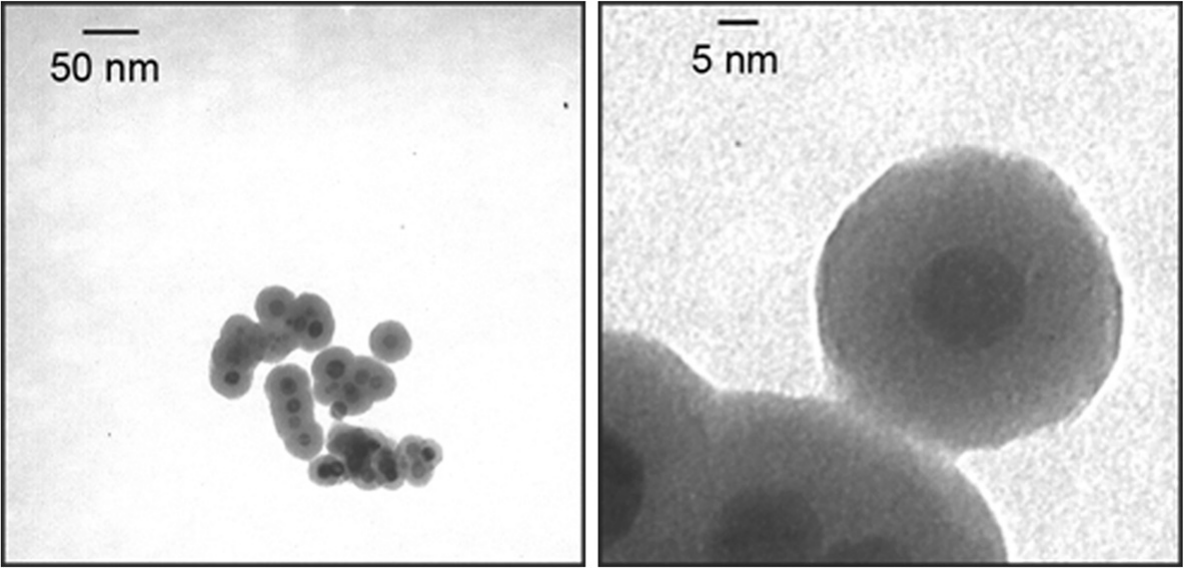
TEM images of synthesized SiMNP at two different magnifications.

High-quality magnetite nanocrystals were synthesized in organic solvents and transferred to water phase using tetramethylammonium hydroxide (TMAOH) as previously described [24]. Next, the water-soluble iron oxide nanoparticles were individually coated with a 10 nm thick silica shell by reaction with tetraethoxysilane (TEOS) in aqueous ammonia and further reacted with the chelating agent ICPTES-NTA and NiCl_2_, [25] resulting in fully armed Ni^2+^ functionalized nanoparticle (NiNTASiMNP). The overall synthesis procedure is summarized in Figure 1. In Figure 2, TEM images show that the final size of SiMNP was 30 ± 3 nm with a magnetic core of 10 ± 1 nm, indicating a homogeneous sample of silica-coated magnetic nanoparticles. Hydrodynamic diameter of SiMNP in ethanol was 80.4 ± 2.3 nm, as determined by dynamic light scattering, which remarkably increased to 128.3 ± 3.7 nm as a consequence of NTA functionalization. Such a difference accounts for a transient loss in nanoparticle stability forming small aggregates after reaction with ICPTES-NTA. However, stable nanoparticle dispersion was recovered after Ni^2+^ chelation, resulting in hydrodynamic size of 89.0 ± 2.8 nm. Zeta-potential (ζ) of SiMNP, NTASiMNP and NiNTASiMNP in water was also investigated. SiMNP and NTASiMNP were negatively charged (-19.11 ± 1.70 mV and -24.42 ± 1.03 mV, respectively), the second being more negative due to the presence of NTA carboxylic groups. The capture of Ni^2+^ ions by the external carboxylic groups of NTA resulted in a more positive charge in NiNTASiMNP (-12.00 ± 1.03 mV). The amount of Ni^2+^ ions captured by NTASiMNP was quantified by inductively coupled plasma optical emission spectrometry (ICP-OES), resulting in 41 ng Ni per mg nanoparticles.

### Immobilization of CP*Sso* on NiNTASiMNP

CP*Sso* protein containing 6 × His tag was produced in *Escherichia coli* and purified by Ni-chelate affinity chromatography, as previously described [21]. Subsequent Ni^2+^ capture by NTA groups of NTASiMNP promoted the active Ni^2+^-NTA affinity-oriented immobilization of His-tagged CP*Sso* (Figure 1). The conjugation reaction was performed at room temperature by incubating NiNTASiMNP (1 mg) with purified CP*Sso* (200 μg). The resulting MNP-CP*Sso* was isolated from unreacted CP*Sso* by centrifugation and washed three times. By measuring the amount of protein found in the supernatants after the enzyme binding process, we determined an amount of CP*Sso* immobilized on nanoparticles of 150 μg per mg of MNP-CP*Sso*. The average CP*Sso* loading was estimated to be about 2 molecules per nanoparticle. To check that the conjugation reaction occurred specifically to Ni^2+^ and His tag, CP*Sso* was incubated in parallel with non-functionalized nanoparticles (NTASiMNP). Then, reaction mixtures were washed, and bound and unbound CP*Sso* determined enzymatically. Non-specifically bound activity was only 4.5% of that bound through the NiNTA linker (Additional file 1: Table S1). These data confirm that CP*SSo* immobilization is mediated by the interaction between Ni^2+^ and His tag. After immobilization, CP*Sso* substantially retained its activity, as shown by comparison of equal amount of free and bound CP*Sso* (data not shown).

### Assessment of the catalytic stability of MNP-CP*Sso*

The catalytic stability of free and conjugated CP*Sso* was analyzed by monitoring the inactivation profiles of the samples incubated in aqueous solution at three different temperatures (25°C, 40°C and 75°C, respectively) and, subsequently, in the presence of organic solvents, *i.e.* dimethylformamide (DMF) or ethanol, at different water ratios.

**Figure 3:**
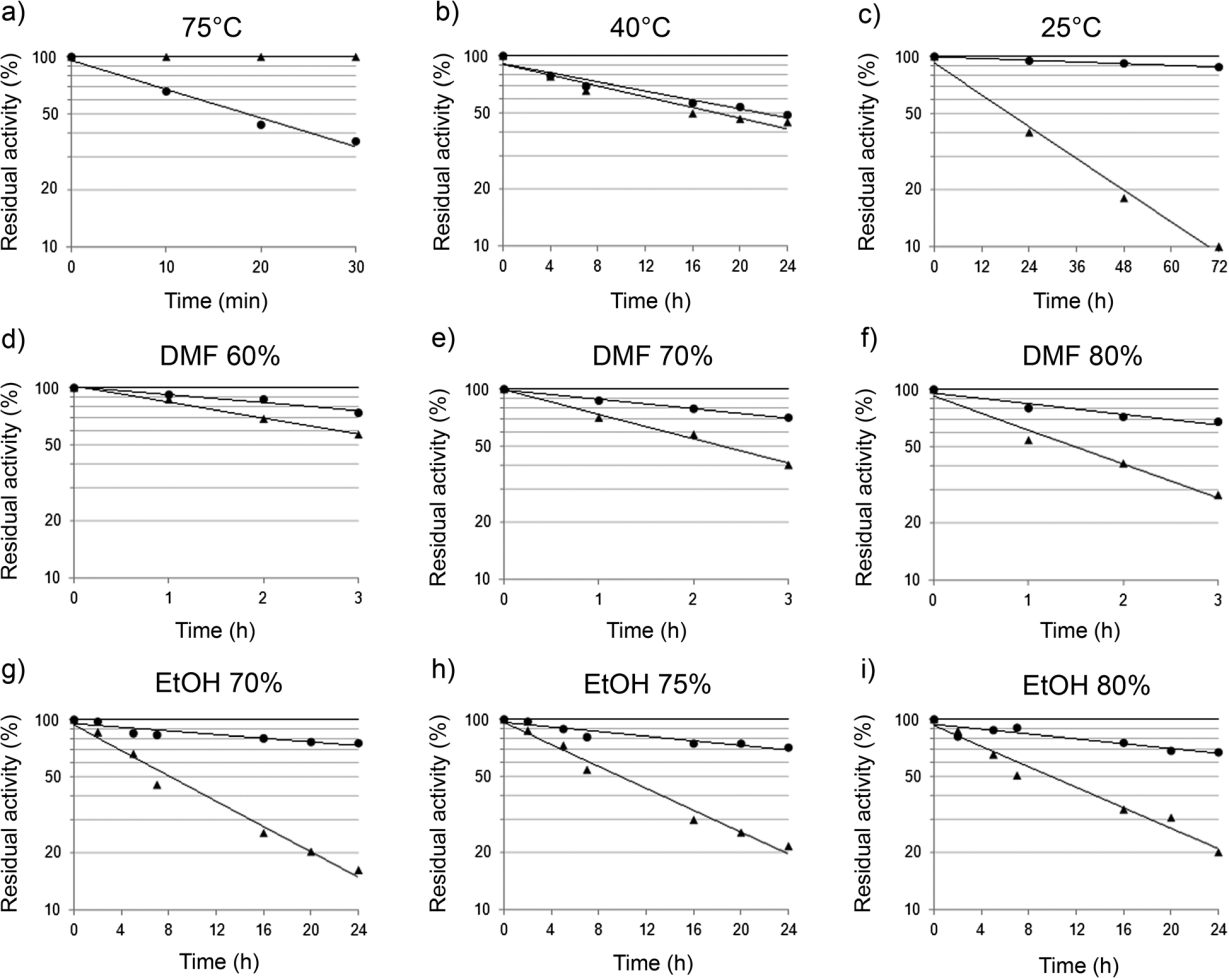
**Inactivation profiles of free (**▲**) and conjugated (**●**) CP*Sso *in aqueous medium (a-c) at 75°C, 40°C and 25°C or in the presence of the indicated DMF (d-f) or ethanol (g-i) concentrations at 40°C.** The enzyme was incubated under shaking at a concentration of 0.6 mg mL^-1^, in 50 mM potassium Mes pH 6.5.

As expected, CP*Sso* was fully active after 30 min incubation at 75°C in aqueous buffer, while MNP-CP*Sso* proved to be less stable (Figure 3a). No significant differences were observed in the stability of free and conjugated enzyme at 40°C (Figure 3b). Functionality profiles obtained at 25°C showed that free CP*Sso* gradually lost its activity resulting in a complete inactivation after 96 h. In such a case, however, enzyme conjugation led to a substantial increase in stability, up to 85% of initial activity being retained after 96 h (Figure 3c). A tentative interpretation of these results is presented in the section Discussion.

The inactivation profiles obtained at 40°C and different concentrations of DMF indicate that CP*Sso* gradually lost its activity by increasing the organic component in the solvent mixture, while the nanobioconiugate retained 80% of residual activity even in the presence of 80% DMF (Figure 3d-f). The results obtained in the presence of ethanol at 40°C and various concentrations are of particular interest. The inactivation profiles show that CP*Sso* had a residual activity of 50% after 6 h, which decreased to 20% after 24 h incubation (Figure 3g-i). However, MNP-CP*Sso* revealed a significantly improved stability in ethanol at the different tested concentrations compared with free CP*Sso*, up to 80-90% of residual activity after 6 h, and 70% after 24 h incubation in 80% ethanol being retained.

### Synthesis of N-blocked amino acids promoted by MNP-CP*Sso*

**Figure 4:**
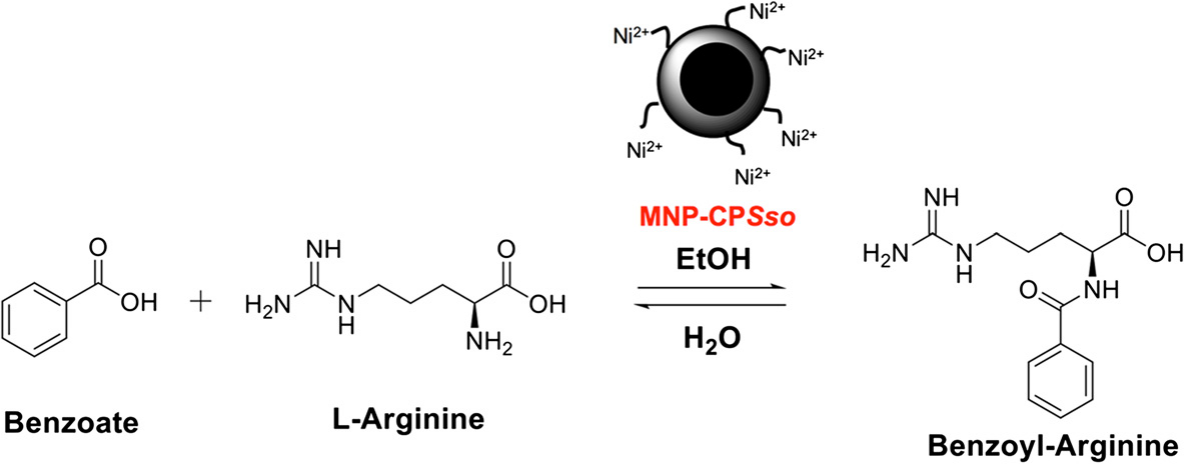
**MNP-CP** ***Sso*-catalyzed synthesis of an N-blocked amino acid.**

**Figure 5:**
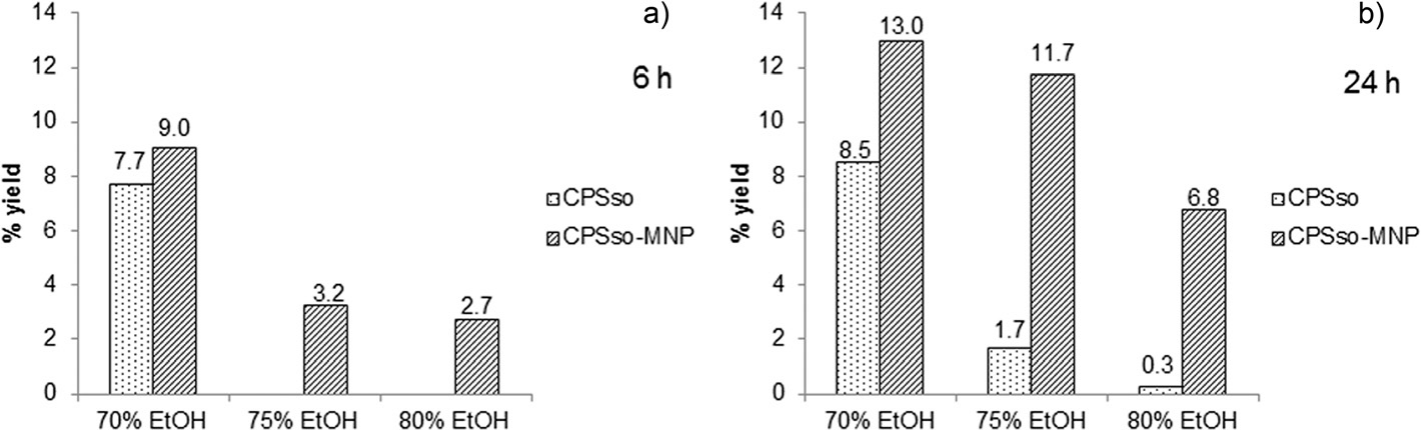
**Synthesis of benzoyl-arginine catalysed by free and conjugated CP*Sso *in the presence of different ethanol concentration.** 0.4 M benzoate, 0.1 M arginine were incubated with 5 U mL^-1^ CP*Sso* at 40°C in 50 mM potassium Mes, pH 6.5, and ethanol at the indicated concentrations. After a 6 h **(a)** or 24 h **(b)** incubation, the amount of benzoyl-arginine synthesized was determined spectrophotometrically.

Preliminary experiments were carried out in order to select the water-cosolvent system and the chemical-physical parameters most suitable to promote the synthesis of *N*-blocked amino acids by CP*Sso,* using arginine and benzoate as a model system (Figure 4). Our results show that the best rate of benzoyl-arginine (BA) synthesis was obtained in 70% ethanol at pH 6.5 and 40°C after 6 h of incubation, using 10 U mL^-1^ CP*Sso*, 0.4 M benzoate and 0.1 M arginine (data not shown). Next, BA synthesis was performed using both free CP*Sso* and MNP-CP*Sso* under the aforementioned optimal conditions. By taking advantage of the remarkable enzyme stability in organic solvent, higher ethanol concentration (75% and 80%) and a longer incubation time (24 h), were also tested. The results obtained after a 6-h incubation (Figure 5) show a similar synthetic rate for free and conjugated enzyme in 70% ethanol. However, at higher ethanol concentrations, only MNP-CP*Sso* could synthesize appreciable amounts of BA, although in much lower yields. Remarkably, when the synthesis was extended to 24 h, higher yields were observed, with the sole exception of unbound CP*Sso* in 70% ethanol. In particular, at 70-75% ethanol, far better yields were attained when using MNP-CP*S*so (Figure 5). Under all adopted conditions, no detectable enzyme activity was found in the reaction mixture after removal of the MNPs (data not shown).

The activity of NiNTASiMNP was retained without significant drop in functionality for three-times recycled uses in comparison with the initial activity, comparable with other systems of similar efficiency [26].

## Discussion

In our ongoing effort in designing innovative approaches for the orientation-controlled conjugation of proteins on metal-based nanoparticles for biotechnological and biomedical applications [5,27-31], we have recently investigated the modulation of the structural properties of the immobilized protein from a biophysical point of view [13]. In the present paper, we report the results of a study conducted on a hyperthermophilic enzyme, namely CP*Sso*, aimed at determining the effect of its immobilization on superparamagnetic nanoparticles on the biological activity of the enzyme.

The nanobioconjugate was obtained by affinity-oriented immobilization of His-tagged CP*Sso* on silica-coated magnetic nanoparticles functionalized with Ni-NTA groups (NiNTASiMNP). The procedure for the synthesis of NiNTASiMNP is illustrated in Figure 1 and the resulting functionalized Fe_3_O_4_@silica core-shell nanoparticles gave a stable dispersion in aqueous environment, as determined by dynamic light scattering analysis.

An important requirement for protein immobilization is that the matrix should provide a biocompatible and inert environment, *i.e.* it should not interfere with the native structure of the protein, which would thereby compromise its biological activity. Thus, we measured the enzymatic activity of free and immobilized CP*Sso* and confirmed that its activity was preserved after conjugation. Next, we evaluated the protein activities in an aqueous milieu to assess the effect of conjugation on the stability of the hyperthermophilic enzyme in dependence of the temperature. Noteworthy, in this respect, is the substantial stability at room temperature of MNP-conjugated CP*Sso*, unlike the free enzyme, which should make it possible to perform transformations without the use of energy for heating or cooling the reaction environment, otherwise needed for maintaining enzyme stability. In a theoretical perspective, this might be rationalized on the basis of the following considerations. As free enzyme undergoes inactivation at 40°C and 25°C, but not at 75°C, in agreement with our previous report [22], this fits with the “cold denaturation” pattern, which is believed to be a general phenomenon in protein chemistry [32]. In the case of free CP*Sso*, optimum stability temperature might be at, or close to 75°C, as significant inactivation was previously observed at 85°C [33]. In contrast, the conjugated enzyme was essentially stable at 25°C and underwent progressive destabilization on increasing temperature, suggestive of a substantial downshift of the stability optimum. Based on enzyme activity decay constants determined at the three temperatures, we assessed an activation enthalpy (ΔH^**‡**^) of enzyme inactivation around 150 kJ · mol^-1^ for the conjugated enzyme, well below values close to 500 kJ · mol^-1^ determined for the free form [33,22]. As the enzyme retains its activity after conjugation, it must retain its overall structure as well. Thus, the dramatic drop in ΔH^‡^ of bound with respect to free enzyme, is suggestive of major conformational constraints of the transition state as a result of immobilization. This is confirmed by an activation entropy value (ΔS^‡^) close to zero (as determined from the data in Figure 3a to c), to be compared with 1.1 kJ · mol^-1^ K^-1^ of the free enzyme [33,22].

It is well-known that enzyme immobilization generally results in increased stability toward the denaturing effect of water-soluble organic solvents [34]. Also, it is believed that such polar solvents first strip water from the surface of the protein, then strongly compete for intramolecular hydrogen bonds, with resulting protein unfolding [35]. In the case of CP*Sso*, the conjugation quite likely limits its conformational flexibility, as above outlined, which may partially prevent the solvent from penetrating the protein interior. The stronger effect of DMF with respect to ethanol fits well with its higher polarity and “denaturing capacity”, as previously defined [36]. Remarkably, we found that at 40°C the conjugated enzyme was even more stable in 80% ethanol than in water.

Improved stability of MNP-CP*Sso* in organic solvent is relevant to possible industrial applications of the enzyme as a biocatalyst in synthetic reactions carried out in organic environment. In particular, also thanks to its broad substrate specificity, CP*Sso* could be an ideal candidate as a biocatalyst for the synthesis of *N*-blocked amino acids in water-cosolvent mixtures following the thermodynamic method. Actually, we previously found that the enzyme is capable of both hydrolyzing N-blocked amino acids in aqueous solution [20], and catalyzing the synthetic reaction in organic solvents [33], irrespective of the amino acid involved (with the sole exception of proline). The presence of organic solvents in solution is in fact deemed necessary to shift the equilibrium toward the formation of the peptide bond, due to their generally low dielectric constant and the consequent prevalence of undissociated forms of carboxylic and amino groups, which are indeed the reactive species in amide linkage formation [37]. It is worthwhile to be mentioned, in this respect, that at 70-75% ethanol, MNP-CP*Sso* catalyzed significant synthesis of benzoyl-arginine (BA) (Figure 5), which is promising for possible industrial applications, whereas the free enzyme’s performance was very poor under the same conditions. The lower levels of BA produced in 75% and 80% compared to 70% ethanol, are likely due to enzyme inhibition by the organic solvent. In fact, it is well known that the kinetic parameters, k_cat_ and K_m_, are strongly affected by organic solvents [38].

## Conclusions

We developed a nanobioconjugate of CP*Sso* immobilized on silica-coated magnetic nanoparticles, which exhibited enhanced stability in aqueous media at room temperature as well as in different organic solvents. The improved stability in ethanol paves the way to possible applications of immobilized CP*Sso*; in particular, our results show that the enzyme could be employed as a biocatalyst for the synthesis of *N*-blocked amino acids. Another potential application might be amino acid racemate resolution, a critical and expensive step in chemical synthesis. This could be accomplished by chemically synthesizing *N*-blocked racemic amino acid mixtures, followed by CP*Sso*-catalyzed selective hydrolysis of the blocking group from the l-enantiomer.

## Methods

### Chemicals and Instrumentation

All chemicals were purchased from Sigma-Aldrich (St. Louis, MO) and used as received. Water was deionized and ultrafiltered by a MilliQ apparatus from Millipore Corporation (Billerica, MA) before use. Transmission electron microscopy (TEM) images were obtained by a Zeiss EM-109 microscope operating at 80 kV, available at the “Centro di Microscopia Elettronica per le Nanotecnologie applicate alla medicina” (CMENA, University of Milan). Dynamic light scattering (DLS) measurements were performed at 90° with a 90 Plus Particle Size Analyzer from Brookhaven Instruments Corporation (Holtsville, NY), working at 15 mW of a solid state laser (λ = 661 nm). Zeta-potential (ζ) measurements were performed on the same instrument, equipped with a couple of AQ-809 electrodes, and analyses were processed by a ZetaPlus software. Viscosity and refractive index of pure water were used to characterize the solvent. Nanoparticles were dispersed in the solvent and sonicated in a S15H Elmasonic apparatus (Elma, Singen, Germany) before analysis. Final sample concentration used for measurements was typically of 5 μg mL^-1^. For ICP-OES analysis, 2 ml of the NiNTASiMNP dispersion (5 mg mL^-1^) was measured in triplicate with Optima 7000 DV ICP-OES (Perkin Elmer).

### Synthesis of NiNTASiMNP

Magnetic nanoparticles (**MNP**) were synthesized in organic solvent by solvothermal decomposition as described in our previous work [30]. The 8 nm iron oxide nanoparticles coated with oleic acid dissolved in chloroform (20 mg, 6.7 mL) were treated with an aqueous solution of tetramethylammonium hydroxide (TMAOH, 0.6 g in 20 ml) overnight under vigorous stirring at room temperature [27,30,39]. The organic solution containing the surfactant was removed by centrifugation at 1000 rpm (3 min). The aqueous solution of TMAOH-coated MNP (20 mg) was concentrated in Amicon Ultra 100.000 MWCO (Millipore) (3000 rpm, 5 min). The resulting nanoparticle dispersion (1.2 mL) was added to a mixture of EtOH (3 mL) and aqueous ammonia (75 μL, 28% by weight), then, a solution of tetraethyl orthosilicate (TEOS) in ethanol (45 μL in 500 μL EtOH) was added dropwise and stirred overnight in order to obtain individual-particle silica coating. The precipitate was collected by magnet, washed three times with EtOH and dried under vacuum. The resulting nanoparticles were dispersed in EtOH (33 mL) (**SiMNP**).

For the synthesis of ICPTES-NTA, a solution of ϵ-(*N*-benzyloxycarbonyl)lysine (**1**) (1.2 g, 4.2 mmol) in 1.5 M NaOH (15 mL) was added dropwise to a solution of bromoacetic acid (2.5 g, 18 mmol) in 1.5 M NaOH (9 mL) at 0°C over 2 h. The mixture was allowed to warm up to 18°C, stirred at this temperature overnight, then at 50°C for 2 h. The cooled reaction mixture was treated dropwise with 1 M HCl (24 mL). The white precipitate was filtered off, washed with 0.1 M HCl (12 mL) and water (2 × 12 mL), then dried under vacuum and then oven-dried at 90°C to give the triacid derivative (**2**) [40].

The product (2 g) was dissolved in 50 mL of MeOH/H_2_O (20:1) under sonication to dissolve the product and, after the addition of a spatula tip of 5% Pd/C, hydrogenated at room temperature and atmospheric pressure. Total hydrogenation of compound was achieved after 90 min as monitored by TLC in CH_3_CN/H_2_O (4:1), visualized by charring with a solution of (NH_4_)_6_Mo_7_O_24_ (21 g), CeSO_4_ (1 g) and 96% H_2_SO_4_ (31 mL) in 500 mL of deionized H_2_O, followed by heating. The catalyst was filtered off, and solvent removed in vacuo. The resulting precipitate was redissolved in H_2_O (20 mL) and EtOH (90 mL) was added. The product crystallizes at 0°C. The crystals were filtered off and dried in vacuo yielding 1.02 g of **3**. Isocyanatopropyltriethoxysilane (ICPTES, 247.4 mg, 0.37 mmol) was dissolved in CHCl_3_ (5 mL) and **3** (0.37 mmol) was then added, followed by a solution of triethylamine (3.34 mmol) in MeOH (10 mL). The resultant solution was stirred overnight. Finally, the solvent was dried under vacuum, yielding 265 mg of **4** (Additional file 1: Scheme S1) [25].

An ICPTES-NTA solution (20 mg in 20 mL EtOH) was added to the SiMNP aqueous dispersion (33 mL, EtOH) and the mixture was heated at 80°C for 3 h under stirring. **NTASiMNP** were recovered after centrifugation and washed twice with EtOH to remove the excess of unreacted ICPTES-NTA. In the last step, NiCl_2_•6H_2_O (95 mg in 4 mL H_2_O) was added to the dispersion of NTASiMNP (20 mg in 6 mL H_2_O). The pH of the solution was adjusted to pH 8.4 with 0.1 N NaOH and the dispersion was sonicated for 30 min. The nanoparticles were washed twice with water by centrifuging at 2500 rpm. The collected washed particles (**NiNTASiMNP**) were dissolved in water and stored at the final concentration of 5 mg mL^-1^. This procedure is briefly outlined in Figure 1, first two steps.

### Affinity purification of histidine-tagged CP*Sso*

Wild-type and mutated histidine-tagged CP*Sso* were purified by Ni-chelate affinity chromatography. *E. coli* cells were grown under shaking at 37°C in LB medium (3 L) containing 0.1% glucose, 50 mg mL^-1^ ampicillin, 25 mg mL^-1^choramphenicol until A_600_ reached 0.8. Induction was then carried out for 3.5 h with 0.4 mM isopropyl β-d-thiogalactopyranoside. Cells were harvested, washed with 50 mM Tris–HCl, pH 8.0, 0.1 M NaCl, 10 mM ZnCl_2_, and resuspended in 3 volumes of 50 mM Tris–HCl, pH 8.0, 1 mM PMSF, 5 mM 2-mercaptoethanol, complete EDTA-free protease inhibitor (1 tablet/50 mL; Roche), 13 units mL^-1^ bovine pancreas DNase (Sigma-Aldrich, St. Louis, MO). Cells were disrupted by sonication and centrifuged at 40000 × g for 30 min. After addition of 10% (by vol.) glycerol and 10 mM imidazole, the supernatant was loaded at a flow rate of 1 mL min^-1^ onto a Ni-NTA Superflow (Qiagen) column (2 mL bed volume) pre-equilibrated with 50 mM potassium Mes, pH 6.0, 10 mM imidazole, 5 mM 2-mercaptoethanol, 10% (by vol.) glycerol. The column was washed with 10 volumes of the same buffer and the enzyme eluted with a 20-mL continuous linear imidazole gradient, 10 mM to 1 M, in the same buffer, and 2-mL fractions were collected (Additional file 1: Figure S1). Fractions displaying the highest purity (at least 95%, as assessed in SDS-PAGE) [41] were concentrated in Amicon Ultra 100.000 MWCO (Millipore) and buffer was exchanged to 50 mM potassium Mes, pH 6.0.

### CP*Sso* activity and protein assays

CP*Sso* activity was assayed continuously using benzoyl-arginine (Bz-Arg) or furylacryloyl-phenylalanine (Fur-Phe) as substrates. The assay mixture contained 50 mM potassium Mes pH 6.5, 0.1 mM Bz-Arg or 0.2 mM Fur-Phe, in a total volume of 1.5 mL. After a 5-min preincubation at 60°C the enzyme was added, and the decrease in absorbance monitored at the same temperature and 239 nm or 330 nm. At these wavelengths, the decrease of the molar absorption coefficient was 6240 M^-1^ cm^-1^ for Bz-Arg and 5750 M^-1^ cm^-1^ for Fur-Phe, respectively. One unit of enzyme activity is defined as the amount that hydrolyzes 1 μmol min^-1^ of substrate under the standard assay conditions. Protein content was determined using the Coomassie Plus Protein Assay Reagent from Pierce and bovine plasma immunoglobulin G as the standard protein.

### CP*Sso* conjugation to NiNTASiMNP

In a plastic tube, NiNTASiMNP (1 mg) were incubated with purified CP*Sso* (200 μg) in 50 mM potassium Mes pH 6.5 in a final volume of 5 mL and the mixture was stirred on an orbital shaker for 15 min at 4°C. CP*Sso*-MNP were isolated from unreacted CP*Sso* by centrifugation at 5000 × g for 3 min and the supernatant was discarded. Nanoparticles were washed three times with 50 mM potassium Mes pH 6.5 (1 ml) and stored in 50 mM potassium Mes pH 6.5 at 4°C. By measuring the amount of protein found in the supernatant after the enzyme binding process, we determined an amount of CP*Sso* immobilized on nanoparticles of 150 μg per mg of MNP-CP*Sso*. The average number of CP*Sso* loaded on MNP-CP*Sso* was estimated to be about 2 molecules per nanoparticle. This procedure is briefly outlined in Figure 1, last step.

### Assessment of CP*Sso* stability in aqueous medium

Free and conjugated CP*Sso* were incubated at the desired temperatures in a thermoshaker under stirring and at a concentration of 60 μg mL^-1^, in 50 mM potassium Mes, pH 6.5. At different times, protein aliquots (about 30 mU) were taken from the mixture and added to 1.5 mL of degassed 50 mM potassium Mes, pH 6.5, 0.1 mM Bz-Arg, already thermostated in a cuvette at 60°C. The reaction was monitored at 239 nm as previously described.

### Assessment of CP*Sso* stability in dimethylformamide

Free and conjugated CP*Sso* were incubated at 40°C in a thermoshaker and at a concentration of 60 μgmL^-1^, in 50 mM potassium Mes, pH 6.5 and the indicated DMF concentrations. At different times, protein aliquots (about 30 mU) were taken from the mixture and added to 1.5 mL of degassed 50 mM potassium Mes, pH 6.5, 0.2 mM Fur-Phe, already thermostated in a cuvette at 60°C. The reaction was monitored at 330 nm as previously described.

### Assessment of CP*Sso* stability in ethanol

Free and conjugated CP*Sso* were incubated at 40°C in a thermoshaker and at a concentration of 60 μg mL^-1^, in 50 mM potassium Mes, pH 6.5, and the indicated ethanol concentrations. At different times, protein aliquots (about 30 mU) were taken from the mixture and added to 1.5 mL of degassed 50 mM potassium Mes, pH 6.5, 0.1 mM Bz-Arg, already thermostated in a cuvette at 60°C. The reaction was monitored at 239 nm as previously described.

### Synthesis of N-blocked amino acids

A solution of 50 mM potassium Mes, pH 6.5, including reaction substrates (sodium benzoate and the amino acid) and organic solvent at the desired concentration was prepared. Final volume was 40 μL including enzyme and nanoparticle. Control samples were prepared with NiNTASiMNP and without CP*Sso*. Samples and controls were incubated in a thermoshaker at the desired temperature for 6 or 24 h. At the end of the respective incubation times, each sample was boiled at 100°C for 15 min to inactivate the enzyme.

### Determination of the products of synthesis

After boiling the samples, quantitative determination of *N*-blocked amino acid produced was carried out by total hydrolysis catalyzed by CP*Sso*. Aliquots were taken from the samples, added to 2.5 mL of degassed 50 mM potassium Mes, pH 6.5, at an appropriate dilution factor (around 1:1000) so that the initial absorbance was not greater than 1.4 absorbance units. Then, the mixture was thermostated for 10 min at 40°C and the assay performed by monitoring the decrease in absorbance at 40°C in the presence of 20–30 mU of CP*Sso* at 242 nm. The decrease in extinction coefficients (Δϵ) for Bz-amino acids was 6058 M^-1^ cm^-1^.

## Abbreviations

CP*Sso*: *Sulfolobus solfataricus* carboxypeptidase; DMF: Dimethylformamide; ICPTES: Isocyanatopropyltriethoxysilane; IPTES-NTA: 15-carboxy-16-(carboxymethyl)-4,4-diethoxy-9-oxo-3-oxa-8,10,16-triaza-4-silaoctadecan-18-oic acid-Ni^2+^; potassium Mes: 2-(N-Morpholino)ethanesulfonic acid potassium salt; MNP: Superparamagnetic iron oxide nanoparticles; NiNTA: Nichel nitriloacetic acid; NiNTASiMNP: Ni^2+^-functionalized silica-coated magnetic nanoparticles; SiMNP: Silica-coated magnetic nanoparticles; TEM: Transmission electron microscopy; TEOS: Tetraethylorthosilicate; TMAOH: Tetramethylammonium hydroxide.

## Competing interests

The authors declare that they no competing interests.

## Authors’ contributions

Conceived and designed the experiments: DP PT SS. Performed the experiments: CB EG JPA MC SS. Analyzed the data: DP MC PT SS. Wrote the paper: DP SS PT. All authors read and approved the final manuscript.

## Supplementary Material

Additional file 1 **Scheme S1.** Synthesis of ICPTES-NTA. **Figure S1.** Progress of *CPSso* purification by Ni-chelate chromatography, as monitored by SDS-PAGE (12% gel). M: molecular weight markers with the respective molecular weights (kDa); CE: crude extract; FT: column flow through; W: column wash; Fr1 to Fr10: individual fractions eluted by an imidazole gradient. For other details, see Materials and Methods. **Table S1.** CPSso binds to MNPs via NiNTA functional groups. The enzyme (ca. 1000 mU) was incubated in the presence of 1 mg of MNP (NiNTASiMNP or NTASiMNP) for 15 min at 4°C under gentle shaking. Then, the mixes were centrifuged and the supernatant (containing the unbound enzyme) removed. Next, the MNPs were washed twice with 50 mM potassium MES, 6.5. Finally, they were resuspended in the same buffer and bound activity determined. Activities in the other fractions (unbound, wash1 and wash 2) were also determined. Click here for file
