# Supplementary material for: Immobilization of carboxypeptidase from Sulfolobus solfataricus on magnetic nanoparticles improves enzyme stability and functionality in organic media
Source: BMC Biotechnol. 2014 Sep 5;14:82. doi: 10.1186/1472-6750-14-82 (PMC4177664; doi:10.1186/1472-6750-14-82)
Supplement: Additional file 1 — Scheme S1. Synthesis of ICPTES-NTA. Figure S1. Progress of CPSso purification by Ni-chelate chromatography, as monitored by SDS-PAGE (12% gel). M: molecular weight markers with the respective molecular weights (kDa); CE: crude extract; FT: column flow through; W: column wash; Fr1 to Fr10: individual fractions eluted by an imidazole gradient. For other details, see Materials and Methods. Table S1. CPSso binds to MNPs via NiNTA functional groups. The enzyme (ca. 1000 mU) was incubated in the presence of 1 mg of MNP (NiNTASiMNP or NTASiMNP) for 15 min at 4°C under gentle shaking. Then, the mixes were centrifuged and the supernatant (containing the unbound enzyme) removed. Next, the MNPs were washed twice with 50 mM potassium MES, 6.5. Finally, they were resuspended in the same buffer and bound activity determined. Activities in the other fractions (unbound, wash1 and wash 2) were also determined. [file 1472-6750-14-82-S1.docx]

**Supplementary information**

**Scheme S1** Synthesis of ICPTES-NTA.


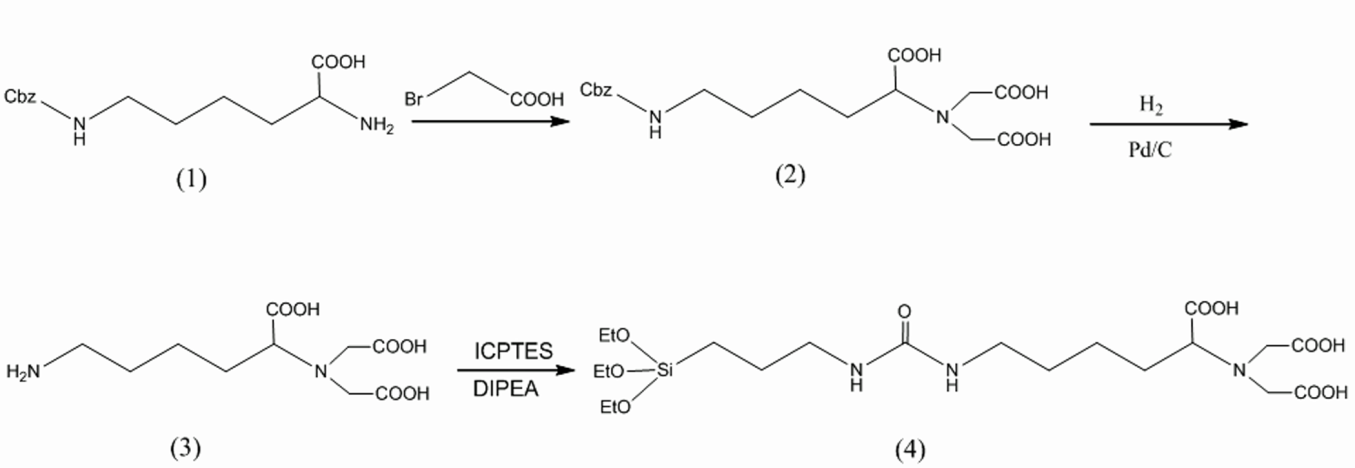


**Fig S1** Progress of *CPSso* purification by Ni-chelate chromatography, as monitored by SDS-PAGE (12% gel). M: molecular weight markers with the respective molecular weights (kDa); CE: crude extract; FT: column flow through; W: column wash; Fr1 to Fr10: individual fractions eluted by an imidazole gradient. For other details, see Materials and Methods.

**
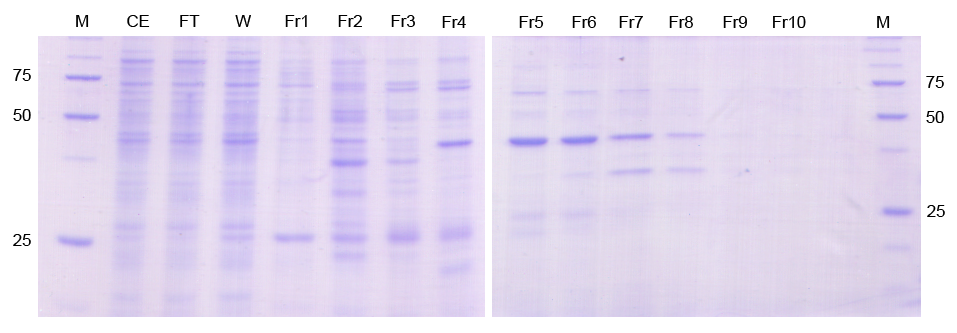
**

**Table S1.**

**CPSso binds to MNPs via NiNTA functional groups.** The enzyme (ca. 1000 mU) was incubated in the presence of 1 mg of MNP (NiNTASiMNP or NTASiMNP) for 15 min at 4°C under gentle shaking. Then, the mixes were centrifuged and the supernatant (containing the unbound enzyme) removed. Next, the MNPs were washed twice with 50 mM potassium MES, 6.5. Finally, they were resuspended in the same buffer and bound activity determined. Activities in the other fractions (unbound, wash1 and wash 2) were also determined.

| **Sample** | **NTASiMNP** | **NiNTASiMNP** |
| --- | --- | --- |
| Total enzyme | 1023 | 1092 |
| Unbound enzyme | 896 | 44 |
| Wash 1 | 25 | b.d. |
| Wash 2 | b.d. | b.d. |
| MNP-bound enzyme | 51 | 815 |

b.d.: below detection.
